# Small Heat Shock Protein Beta-1 (HSPB1) Is Upregulated and Regulates Autophagy and Apoptosis of Renal Tubular Cells in Acute Kidney Injury

**DOI:** 10.1371/journal.pone.0126229

**Published:** 2015-05-11

**Authors:** Tatsuki Matsumoto, Madoka Urushido, Haruna Ide, Masayuki Ishihara, Kazu Hamada-Ode, Yoshiko Shimamura, Koji Ogata, Kosuke Inoue, Yoshinori Taniguchi, Takafumi Taguchi, Taro Horino, Shimpei Fujimoto, Yoshio Terada

**Affiliations:** 1 Department of Endocrinology, Metabolism and Nephrology, Kochi Medical School, Kochi University Kohasu, Oko-cho, Nankoku, Japan; 2 Center for Innovative and Translational Medicine, Kochi Medical School, Kochi University, Kohasu, Oko-cho, Nankoku, Japan; 3 Department of Pediatrics, Kochi Medical School, Kochi University, Kohasu, Oko-cho, Nankoku, Japan; Tokushima University Graduate School, JAPAN

## Abstract

**Background:**

Heat shock protein beta-1 (HSPB1, also known as HSP27) is a small heat shock protein involved in many cellular processes and reportedly protects cells against oxidative stress. Autophagy protects cells from many types of stress and is thought to play a key role in preventing stress in acute kidney injury (AKI). However, little is known about the role of HSPB1 in autophagy and apoptosis in the pathogenesis of AKI.

**Methods:**

We used a rat ischemia/reperfusion AKI model and cultured renal tubular cells as an *in vitro* model. To elucidate the regulation of HSPB1, we evaluated the promoter activity and expression of HSPB1 in normal rat kidney (NRK)-52E cells in the presence of H_2_O_2_. To examine the regulation of autophagy by HSPB1, we established NRK-light chain 3 (NRK-LC3) cells that were stably transfected with a fusion protein of green fluorescent protein and LC3.

**Results:**

The results of immunohistological examination showed that HSPB1 was expressed in proximal tubule cells after AKI. Real-time quantitative reverse transcription-polymerase chain reaction and western blot analysis showed that HSPB1 messenger RNA and protein expression were upregulated 6–72 h and 12–72 h, respectively, after ischemia/reperfusion injury. HSPB1 promoter activity as well as messenger RNA and protein expression indicated dose-dependent induction by H_2_O_2_. HSPB1 overexpression-induced autophagy in NRK-LC3 cells under normoxic conditions was confirmed with confocal microscopy, which revealed the presence of LC3-positive granules. Furthermore, H_2_O_2_-induced autophagy was inhibited by the transfection of small interfering RNAs for HSPB1. Overexpression of HSPB1 reduced BAX activation and H_2_O_2_-induced apoptosis, as measured by caspase 3 activity and terminal deoxynucleotidyl transferase deoxyuridine triphosphate nick end labeling assay.

**Conclusions:**

We showed that HSPB1 expression increased during oxidative stress in AKI. Incremental HSPB1 expression increased autophagic flux and inhibited apoptosis in renal tubular cells. These results indicate that HSPB1 upregulation plays a role in the pathophysiology of AKI.

## Introduction

Acute kidney injury (AKI), often resulting from ischemic, toxic, and septic insults, is a common disorder with a high morbidity and mortality [1]. The major morphologic changes in ischemic AKI include the effacement and loss of the proximal tubule brush border, patchy loss of tubular cells, areas of focal proximal tubular dilation, apoptosis, necrosis, and inflammation [1]. Ischemia/reperfusion (I/R) injury is among the most common causes of AKI, and the underlying pathogenesis involves injury to nephron segments, both from the ischemia itself and from the mechanism of survival or death under oxidative stress [2, 3]. Proximal renal tubular cells along the nephron segments are particularly sensitive to hypoxia because of their high rates of adenosine triphosphate consumption. Mitochondrial damage is one of the most important factors influencing the survival of proximal tubular cells [2, 3].

Autophagy, a lysosomal degradation pathway, is an essential cellular adaptation for avoiding genotoxic stress, oxidative stress, accumulation of misfolded proteins, nutrient deprivation, and many other types of stress. Studies of the role of autophagy in AKI have reported both beneficial and detrimental effects [4, 5, 6, 7]. It has also been reported that light chain 3 (LC3), a mammalian ATG8 homologue, is essential for autophagy [8]. A revolutionary approach to monitoring autophagy is the use of a green fluorescent protein-LC3 (GFP-LC3) fusion protein to visualize autophagosomes *in vivo* [8]. Several studies, including ours, have demonstrated close connections between autophagy and mitochondrial turnover [9–11]. Indeed, autophagy removes mitochondria that contain damaged components (mitophagy).

Several novel proteins, including heat shock protein (HSP) beta-1 (HSPB1, also known as HSP27), reportedly regulate autophagy. HSPs form a protein superfamily and respond to heat and other physiological stress [12, 13]. HSP27 (in humans and rats) and *HSP25* (in mice) belong to the small HSP (sHSP) subfamily, which contains proteins characterized by low molecular mass and conserved COOH-terminal domains (the α-crystallin domain). HSP27 and *HSP25* are ubiquitous sHSPs, the expression of which is induced in response to a wide variety of unfavorable physiological and environmental conditions. These sHSPs protect cells from otherwise lethal circumstances, mainly through their involvement in pathways of cell death such as necrosis and apoptosis [14]. However, little is known about the role of HSPB1 in autophagy and apoptosis in AKI pathogenesis.

The aim of this study was to examine HSPB1-mediated signaling in relation to autophagy and apoptosis in renal tubular cells. Our data demonstrate that autophagy is induced in renal tubules through the HSPB1 pathway in AKI.

## Materials and Methods

### Induction of AKI

Experiments were performed on male Sprague-Dawley rats (Saitama Experimental Animal Supply, Saitama, Japan) weighing 150–200 g. The rats were anesthetized with sodium pentobarbital (30 mg/kg) via intraperitoneal injection. The left renal artery was occluded with Sugita aneurysm clips (Mizuho Ikakogyo, Tokyo, Japan) for 60 min, after which the clamps were removed and the incisions were closed to induce ischemic injury. The animals (n = 5 per time point) were then sacrificed at various time points (0, 6, 12, 24, 48, and 72 h after surgery), and their left kidneys were processed for histology and to isolate protein and RNA as previously described [15, 16]. Age- and weight-matched control rats (n = 3) received a sham operation and were sacrificed at 0, 6, 12, 24, 48, and 72 h after surgery. This study was carried out in strict accordance with the recommendations of the Guide for the Care and Use of Laboratory Animals of the University of Kochi. The experimental protocol was approved by the Committee on the Ethics of Animal Experiments of the University of Kochi (Permit Number: G-00098). All surgeries were performed under sodium pentobarbital anesthesia, and all efforts were made to minimize the suffering of the animals.

### Cell culture, plasmids, small interfering RNAs, and reagents

Normal rat kidney (NRK)-52E cells (a renal proximal tubular cell line) obtained from the American Type Culture Collection (Manassas, VA) were grown in Dulbecco’s modified Eagle’s medium (DMEM; Gibco Laboratories, Grand Island, NY) supplemented with 50 IU/mL penicillin and 10% heat-inactivated fetal calf serum (Gibco Laboratories, Grand Island, NY) [15]. For experiments involving H_2_O_2_, 200, 400, or 600 μM H_2_O_2_ was added to the NRK-52E cells for 4 h. For starvation experiments, NRK-52E cells were incubated in HANKS medium without serum for 24 h. Expression vectors encoding wild-type human HSPB1 were obtained from OriGene Technologies, Inc. (Rockville, MD) and transfected into the NRK-52E cells via electroporation (360V, 960 μFD), as previously described [15]. Small interfering RNAs (siRNAs) specific for HSPB1 and LC3 and control-scrambled siRNAs were purchased from Life Technologies (Gaithersburg, MD). NRK-52E cells were transfected with the siRNAs via lipofection, as previously described [15]. Rapamycin was obtained from Focus Biomolecules (Plymouth meeting, PA) and bafilomycin A was obtained from LC Laboratories (Woburn, MA). H_2_O_2_ was obtained from Sigma-Aldrich Japan K.K. (Tokyo, Japan). All other chemicals were purchased from Funakoshi (Tokyo, Japan).

### Isolation and histological examination of kidney tissue

Kidney tissue was embedded in paraffin wax, and 5-μm sections were stained with a periodic acid-Schiff staining kit, as previously described [17]. After preincubation of the sections with blocking peptides (10 μg/mL), immunohistochemical staining was performed with a streptavidin-biotin technique and antibodies specific to HSPB1 (Cell Signaling Technology Inc., Danvers, MA), anti-LC3 (MBL, Nagoya, Japan), and anti-aquaporin-1 (anti-AQP1, a marker of proximal tubules; Santa Cruz Biotechnology, Inc., Dallas, TX; catalog #sc-25287), as previously described [15, 17].

### Western blot analysis

Homogenized total renal tissue or NRK-52E cells were lysed in a sodium dodecyl sulfate sample buffer (50 mM HEPES, pH 7.5; 150 mM NaCl; 1.5 mM MgCl2; 1 mM ethylene glycol tetraacetic acid; 10% glycerol; 1% Triton X-100; 1 μg/mL aprotinin; 1 μg/mL leupeptin; 1 mM phenylmethylsulfonyl fluoride; and 0.1 mM sodium orthovanadate) at 4°C [18]. Protein (50 μg/sample) was transferred to a nitrocellulose membrane and probed with the appropriate primary antibodies (anti-LC3 [MBL], anti-HSPB1 and phospho-specific anti-S82-HSPB1 [Cell Signaling Technology Inc.], anti-actin [Santa Cruz Biotechnology, Inc.; catalog #sc-10731], caspase 3 [Cell Signaling Technology Inc.; catalog #9665], a 1:1000 dilution of cleaved caspase 3 [Cell Signaling Technology Inc.; catalog #9664], activated Bax [6A7; Trevigen, Inc., Gaithersburg, MD; catalog #2281-MC-100], CHOP [Cell Signaling Technology Inc.; catalog #785324], or total Bax [5B7; Pharmingen; catalog #556467]). The primary antibodies were detected using horseradish peroxidase-linked anti-rabbit immunoglobulin G and visualized using an Amersham ECL system (Amersham Corp., Arlington Heights, IL). We performed densitometric analysis of LC3-II:actin and SQSTM1:actin.

### Real-time quantitative polymerase chain reaction

We performed real-time quantitative reverse transcription-polymerase chain reaction (RT-PCR), as previously described [19], to analyze RNA extracted from the kidneys with TRI-REAGENT (Life Technologies). Total RNA samples (1 μg) were reverse-transcribed, and RT-PCR was performed to quantify HSPB1 gene expression with an ABI LightCycler Real-Time PCR System (ABI, Los Angeles, CA). RT-PCR of glyceraldehyde-3-phosphate dehydrogenase served as a positive control. A three-step PCR was performed for 35 cycles. The samples were denatured at 94°C for 30 s, annealed at 58°C for 30 s, and extended at 72°C for 30 s. The primers were purchased from Applied Biosystems Inc. (Los Angeles, CA). The efficiency curves were generated and the ratios calculated using the delta-delta Ct method.

### Transient transfection and luciferase assay

The human HSPB1 promoter (-2.3 kb) luciferase plasmid was obtained from SwitchGear Genomics (Menlo Park, CA). NRK-52E cells were transfected via electroporation with plasmid DNA (10 μg), and luciferase activity was measured 48 h later [19, 20]. A β-galactosidase reporter construct was used for the normalization of transfection. We established NRK-52E cells that were stably transfected with a GFP-LC3 fusion protein as a marker of autophagy [21]. The formation of GFP-positive autophagosomes indicated autophagy in these cells. We confirmed transfection efficiency by immunostaining transfected cells with the HSPB1 antibody.

### Scanning laser confocal immunofluorescence microscopy and electron microscopy

NRK-52E cells and kidney sections were fixed with 2% paraformaldehyde in phosphate-buffered saline for 1 h and processed for confocal microscopy imaging, as previously described [15]. Double-stained NRK-52E cells and kidney tissues were fixed in phosphate-buffered saline containing 2% paraformaldehyde and 0.1% glutaraldehyde for 2 h. The samples were embedded in Epon-Araldite resin (Canemco Inc., Québec, Canada), and ultrathin sections were cut according to standard procedures and examined under a Philips EM420 electron microscope [21]. We also counted the number of LC3 puncta-positive cells using confocal microscopy. Cells containing more than 5 LC3 puncta were considered LC3 positive. The average numbers of LC3 puncta per cell in control and HSPB1 transfected cells were 2.5 ± 0.5 and 11.6 ± 2.4, respectively.

### Caspase 3 and terminal deoxynucleotidyl transferase-mediated deoxyuridine triphosphate nick end labeling assays and measurement of released cytochrome c

A Caspase 3 Fluorometric Protease Assay Kit (MBL) was used to measure caspase 3 activity, as previously described [19]. The cell lysates were then incubated using the same amount of reaction buffer and 50 mM caspase 3 substrate for 2 h at 37°C. Fluorescence was monitored at an excitation wavelength of 400 nm and an emission wavelength of 505 nm. An apoptosis deoxynucleotidyl transferase-mediated deoxyuridine triphosphate nick end labeling (TUNEL) Kit II (MBL) was used to stain TUNEL-positive cells as described elsewhere [21]. A cytochrome c release assay kit (Abcam #ab5311) was used according to the manufacturer’s instructions. The cell lysate was separated into mitochondria and cytosol fractions. Western blot analysis was performed with the cytosol fractions and anti-cytochrome c antibody (Abcam k257-100-5).

### Statistical analysis

The results are summarized as means ± standard error of the mean. We used non-parametric statistical tests (Mann Whitney test for unpaired data and Wilcoxon matched pairs test for paired data) instead of the Student’s *t* test. A P-value of <0.05 was considered statistically significant.

## Results

### *HSPB1* gene expression after ischemic AKI *in vivo*

**Fig 1:**
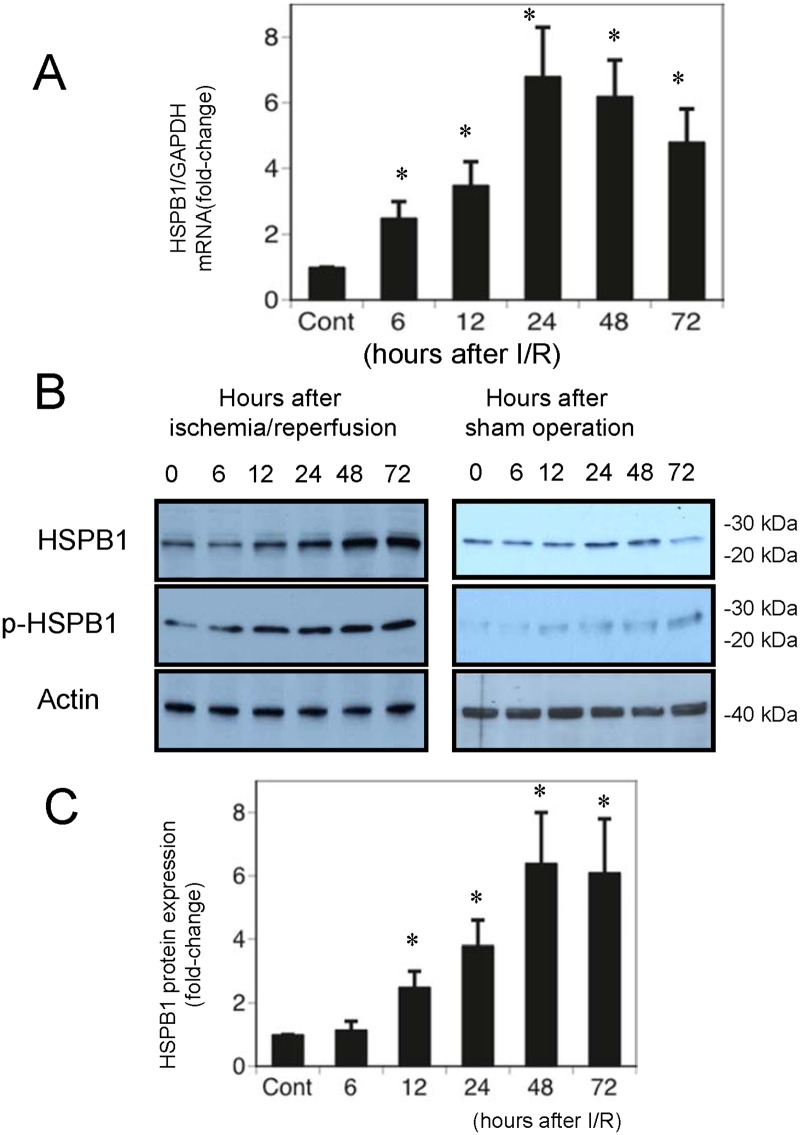
Ischemic/reperfusion (I/R) acute kidney injury (AKI) upregulates messenger RNA (mRNA) and protein expression of heat shock protein beta-1 (HSPB1). The left renal artery was clamped for 60 min, and the kidneys were excised 6, 12, 24, 48, and 72 h after reperfusion. Sham-operated rats killed at 0 h served as controls. (A) HSPB1 mRNA expression was measured with real-time quantitative polymerase chain reaction and normalized to levels of glyceraldehyde-3-phosphate dehydrogenase mRNA. (B) Protein (50 μg) from renal tissue extracts was separated on sodium dodecyl sulfate-polyacrylamide gel electrophoresis (SDS-PAGE) gels. HSPB1 and phospho-HSPB1 were detected with western blot analysis. Actin served as the loading control. (C) Quantitative densitometry was performed for HSPB1. Data are presented as means ± standard error of the mean (SEM); n = 5; *P < 0.05 vs. control rats.

To examine changes in messenger RNA (mRNA) expression of HSPB1 during I/R AKI, we conducted real-time quantitative PCR analysis on rat kidney mRNA. The left renal artery was clamped for 60 min, and the kidney was excised from animals killed 6, 12, 24, 48, and 72 h after reperfusion. Kidneys from sham-operated rats were used as controls at 0 h. Real-time quantitative PCR analysis showed that compared with those in control kidney tissue extracts, *HSPB1* mRNA levels in AKI model extracts began increasing significantly 6 h after I/R injury (Fig 1A).

### HSPB1 protein expression after ischemic AKI *in vivo*

Western blot analyses performed on kidney extracts showed that compared with control extracts, AKI model rat extracts had markedly higher HSPB1 protein expression 12–72 h after I/R injury (Fig 1B and 1C). We also examined phosphorylation of Ser 82-HSPB1 after I/R injury. Western blot analyses of kidney extracts showed that Ser 82-HSPB1 protein expression in AKI model rats 12–72 h after I/R injury was markedly increased compared with that in control extracts (Fig 1B). In rats killed 0, 6, 12, 24, 48, and 72 h after the sham operation, HSPB1 protein expression was slightly upregulated 24 and 48 h post-surgery. However, compared with the sham operation, I/R injury significantly increased HSPB1 expression.

### Immunohistochemical examination of HSPB1 and LC3 expression in ischemic AKI

**Fig 2:**
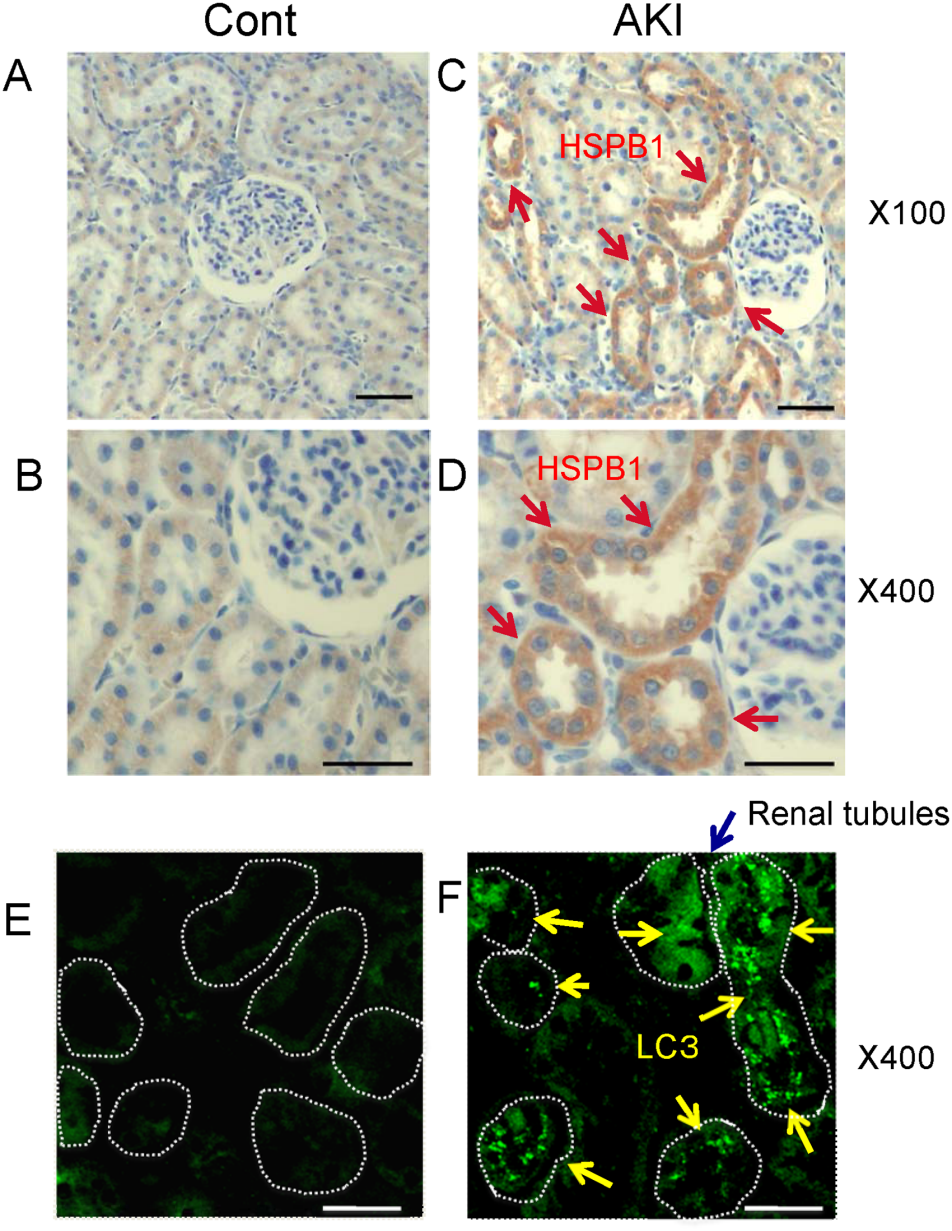
Immunohistochemical analysis showed that HSPB1 and light chain 3 (LC3) expression increased in proximal tubules after I/R AKI. Immunohistochemical analysis of HSPB1 expression in (A, B) the renal cortex of a control kidney (magnification, 100× and 400×) and (C) the renal cortex of a kidney 24 h after I/R injury (magnification, 100×). (D) High-power view of the renal cortex of a kidney 24 h after I/R injury (stained with anti-HSPB1 antibody; magnification, 400×). (E) Immunohistochemical analysis of LC3 expression in the renal cortex of a control kidney (magnification, 400×). (F) Immunohistochemical analysis of LC3 expression in the renal cortex 24 h after I/R injury (magnification, 400×). All scale bars represent 50 μm.

**Fig 3:**
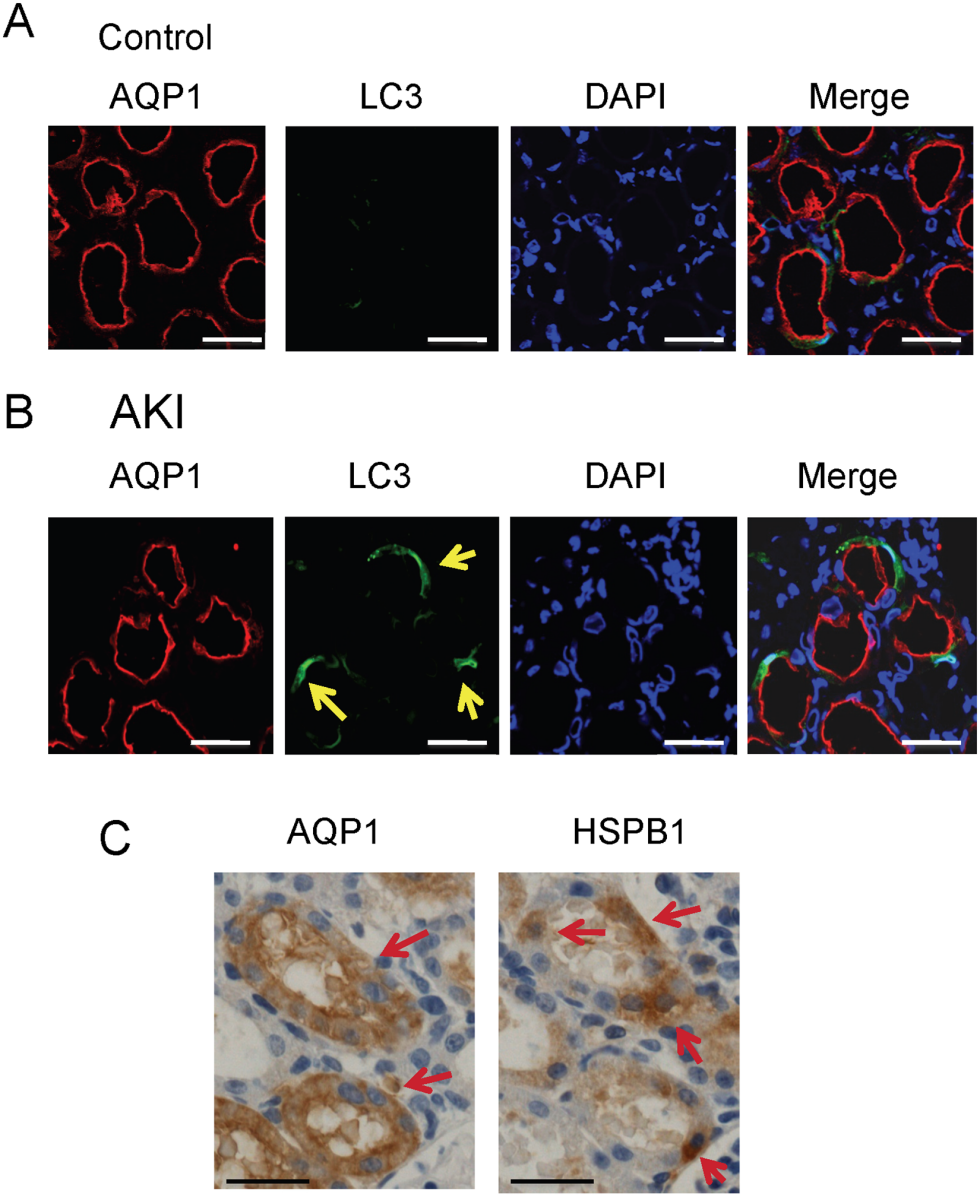
HSPB1 and LC3 are expressed in the same proximal tubules after I/R AKI. Immunohistochemical analysis of aquaporin-1 (AQP1) and LC3 expression in the renal cortex of (A) a control kidney and (B) a kidney 24 h after I/R injury. Confocal microscopy examination showed that AQP1 and LC3 are present in the same renal tubules 24 h after I/R injury. (C) Immunohistochemical analysis of AQP1 and HSPB1 expression in continuous sections in the renal cortex 24 h after I/R injury (magnification, 400×). All scale bars represent 50 μm.

Immunohistological studies of HSPB1 expression showed low levels of HSPB1 in the renal cortical tubules of control rats (Fig 2A and 2B). Comparatively higher levels of HSPB1 expression were found in the renal cortical tubules 24 h after I/R injury (Fig 2C). Moreover, higher magnifications showed that HSPB1 expression was mainly localized in the cytoplasm (Fig 2D). Immunohistological expression of LC3 was found in the renal cortical tubules 24 h after I/R injury (Fig 2F). In contrast, only low levels of LC3 were detected in the renal cortical tubules of control rats (Fig 2E). We used the anti-AQP1 antibody as a marker of proximal tubules [16, 17] and performed confocal microscopy experiments to show that AQP1 and LC3 were present in the same renal tubules. LC3 was stained in AQP1-positive proximal tubular cells (Fig 3A and 3B). Hence, HSPB1 and LC3 were primarily expressed in the renal proximal tubules 24 h after I/R injury. We also observed that AQP1 and HSPB1 were stained in the same renal tubules when continuous sections were immunostained (Fig 3C).

### Increased HSPB1 promoter activity and elevated mRNA and protein expression in H_2_O_2_-treated NRK-52E cells *in vitro*

**Fig 4:**
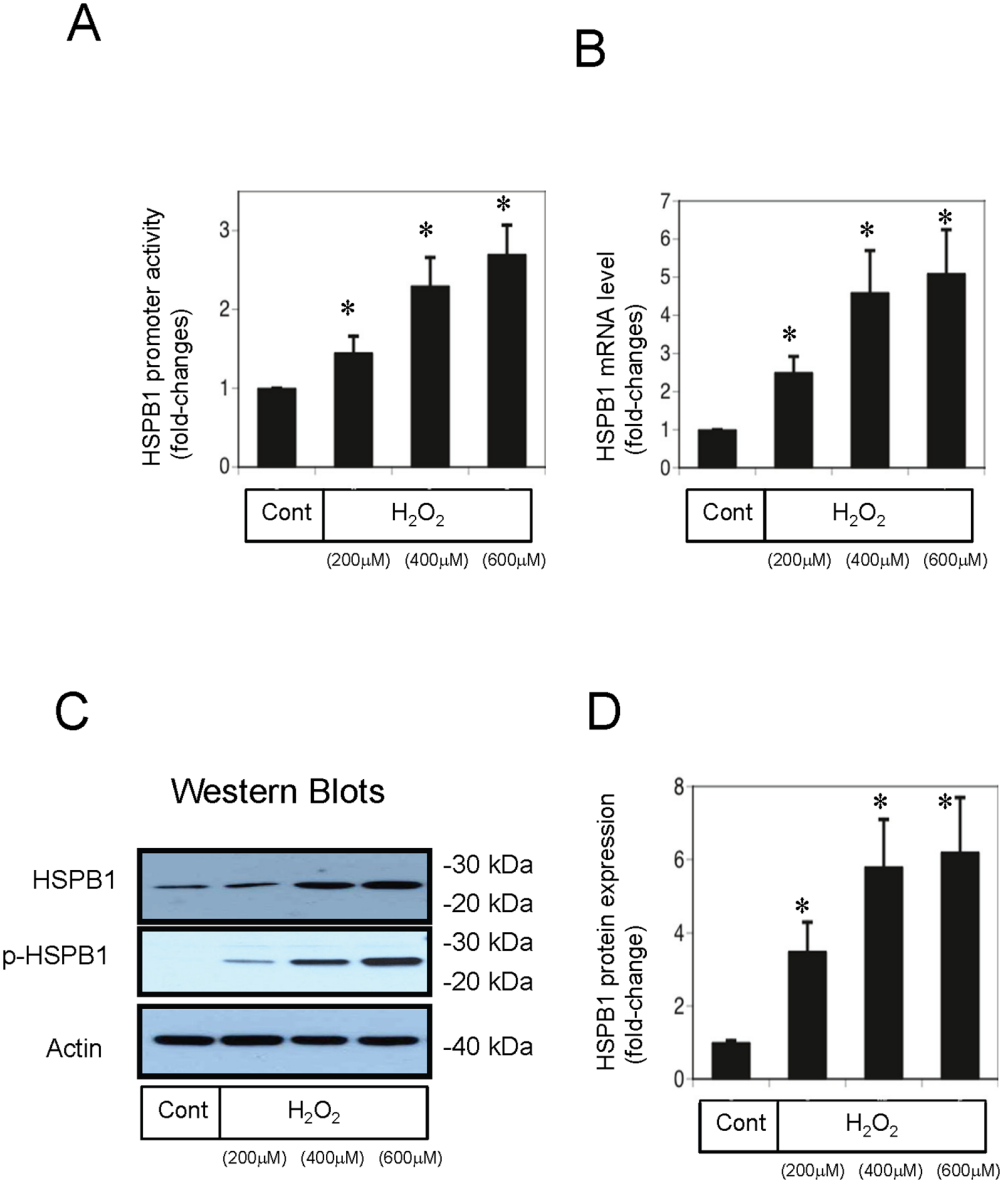
HSPB1 promoter activity, mRNA levels, and protein expression are increased in normal rat kidney (NRK)-52E cells under oxidative stress. NRK-52E cells were exposed to H_2_O_2_ (200, 400, or 600 μM) for 4 h, and (A) HSPB1 promoter activity was measured with luciferase assays, (B) HSPB1 mRNA levels were measured with polymerase chain reaction, and (C) HSPB1 protein and phospho-HSPB1 protein expression was measured with western blot analysis. (D) Densitometric analysis of HSPB1 protein expression after H_2_O_2_ exposure (200, 400, or 600 μM). Data are presented as means ± SEM, n = 5; *P < 0.05 vs. control cells.

Examination of HSPB1 promoter activity, mRNA levels, and protein expression showed that 4 h of exposure to H_2_O_2_ significantly increased HSPB1 promoter activity in cultured NRK-52E cells compared with control cells (Fig 4A). Furthermore, exposure of NRK-52E cells to H_2_O_2_ significantly increased *HSPB1* mRNA expression to 5.1-fold that of control cells (Fig 4B). Exposure to 200, 400, and 600 μM H_2_O_2_ markedly increased HSPB1 protein expression to 3.6-, 5.9-, and 6.2-fold that of control cells, respectively (Fig 4C and 4D). We also examined the phosphorylation of Ser 82-HSPB1 after H_2_O_2_ exposure and observed that exposure to 200, 400, and 600 μM H_2_O_2_ markedly increased phosphorylated Ser 82-HSPB1 protein expression (Fig 4C).

### Modulation of autophagy in NRK-LC3 cells via manipulation of HSPB1 levels

**Fig 5:**
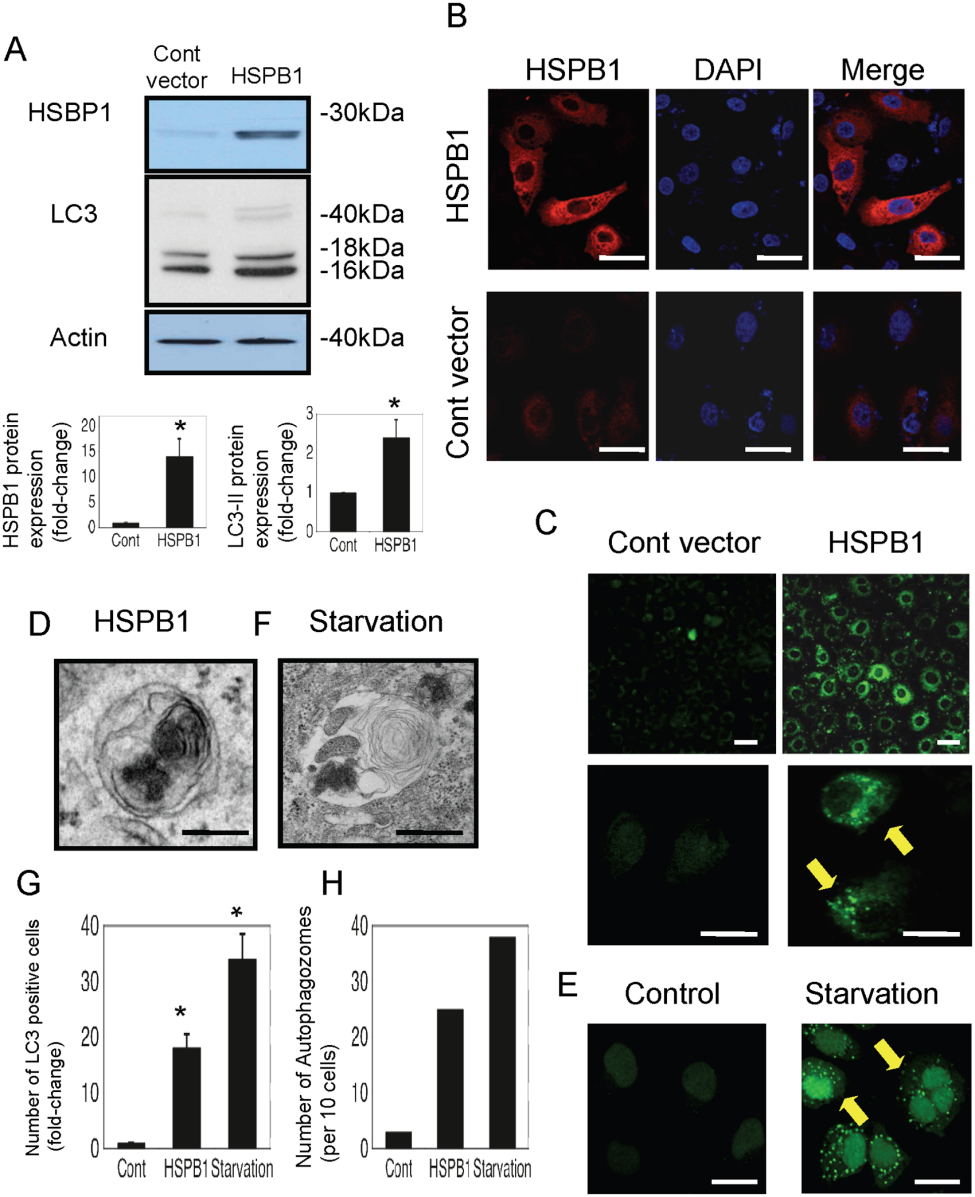
LC3 expression is increased by the overexpression of HSPB1 in NRK-LC3 cells. (A) Western blotting of HSPB1 and LC3 in control or HSPB1-overexpressing NRK-52E cells. Densitometric analysis of HSPB1 protein and LC3-II protein expression after transfection. Data are presented as means ± SEM, n = 5; *P < 0.05 vs. control vector. (B) Immunofluorescence examination of HSPB1 expression in control vector-transfected and HSPB1 expression vector-transfected NRK-52E cells. All scale bars represent 20 μm. (C) Confocal microscopy examination of green fluorescent protein-positive autophagosomes in NRK-LC3 cells transfected with control or HSPB1 expression vectors. All scale bars represent 20 μm. (D) Electron micrograph of autophagosomes in cells that overexpressed HSPB1. Scale bar represents 200 nm. (E) Confocal micrograph of green fluorescent protein-positive autophagosomes in NRK-LC3 cells incubated under starvation conditions. All scale bars represent 20 μm. (F) Electron micrograph of autophagosomes in cells under starvation conditions. Scale bar represents 200 nm. (G) The number of LC3-positive cells that overexpressed HSPB1 or the control vector under starvation conditions, determined with confocal microscopy. Data are presented as means ± SEM, n = 5; *P < 0.05 vs. control cells. (H) The number of autophagosomes in 10 cells that overexpressed HSPB1 and the control vector, determined with electron microscopy.

Next, we transiently transfected NRK-LC3 cells (NRK-52E cells that stably express GFP-LC3) with control or HSPB1 expression vectors to examine the functional role of HSPB1 in autophagy. The transfection efficiency was confirmed with immunofluorescence study and western blot analysis. As shown in Fig 5A, HSPB1 protein expression was markedly increased by the transfection of the HSPB1 expression vector. In accordance with the results of the immunofluorescence study, HSPB1 staining in cells transfected with the HSPB1 expression vector was markedly higher than that in cells transfected with the control vector (Fig 5B).

**Fig 6:**
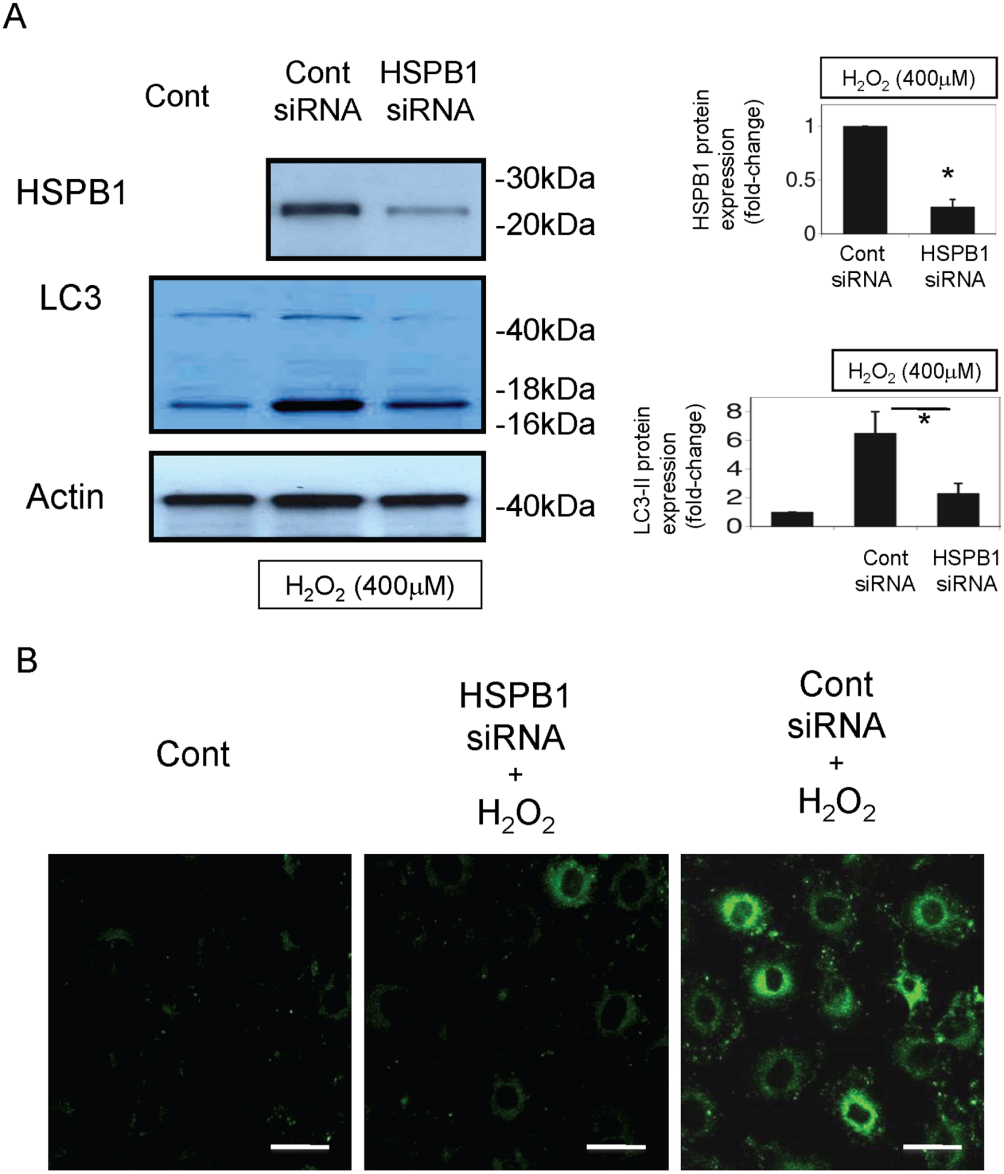
LC3 expression is reduced by HSPB1 small interfering RNA (siRNA) in NRK-LC3 cells. (A) Western blotting of HSPB1 and LC3 in control or HSPB1 siRNA-transfected NRK-52E cells incubated with 400 μM H_2_O_2_ for 4 h. Densitometric analysis of HSPB1 protein and LC3-II protein expression after H_2_O_2_ exposure (400 μM). Data are presented as means ± SEM, n = 5; *P < 0.05 vs. control siRNA. (B) Confocal micrograph of green fluorescent protein-positive autophagosomes in HSPB1 siRNA-transfected NRK-LC3 cells incubated with 400 μM H_2_O_2_. All scale bars represent 20 μm.

Western blot analysis showed that the expression of the autophagy marker LC3-II (16 kDa) in cells that overexpressed HSPB1 was markedly higher than that in control cells (Fig 5A). The GFP-LC3 fusion protein was detected at approximately 40–50 kDa and was slightly induced. Furthermore, we observed many GFP-positive autophagosomes in HSPB1-overexpressing cells using confocal microscopy (Fig 5C). Autophagosome formation was also observed in electron micrographs of HSPB1-overexpressing cells (Fig 5D). We observed many GFP-positive autophagosomes with confocal microscopy and autophagosomes with electron microscopy in cells under starvation conditions (Fig 5E and 5F). The number of autophagosomes and LC3-positive NRK-LC3 cells was significantly higher in cells transfected with the HSPB1 expression vector compared with those transfected with the control (Fig 5G and 5H). In contrast, compared with control cells, NRK-LC3 cells transfected with HSPB1 siRNA and incubated with H_2_O_2_ for 4 h showed significantly lower LC3-II (16 kDa) expression and smaller numbers of GFP-positive autophagosomes after H_2_O_2_ exposure (Fig 6A and 6B).

### HSPB1 increased autophagic flux in NRK-52E cells

**Fig 7:**
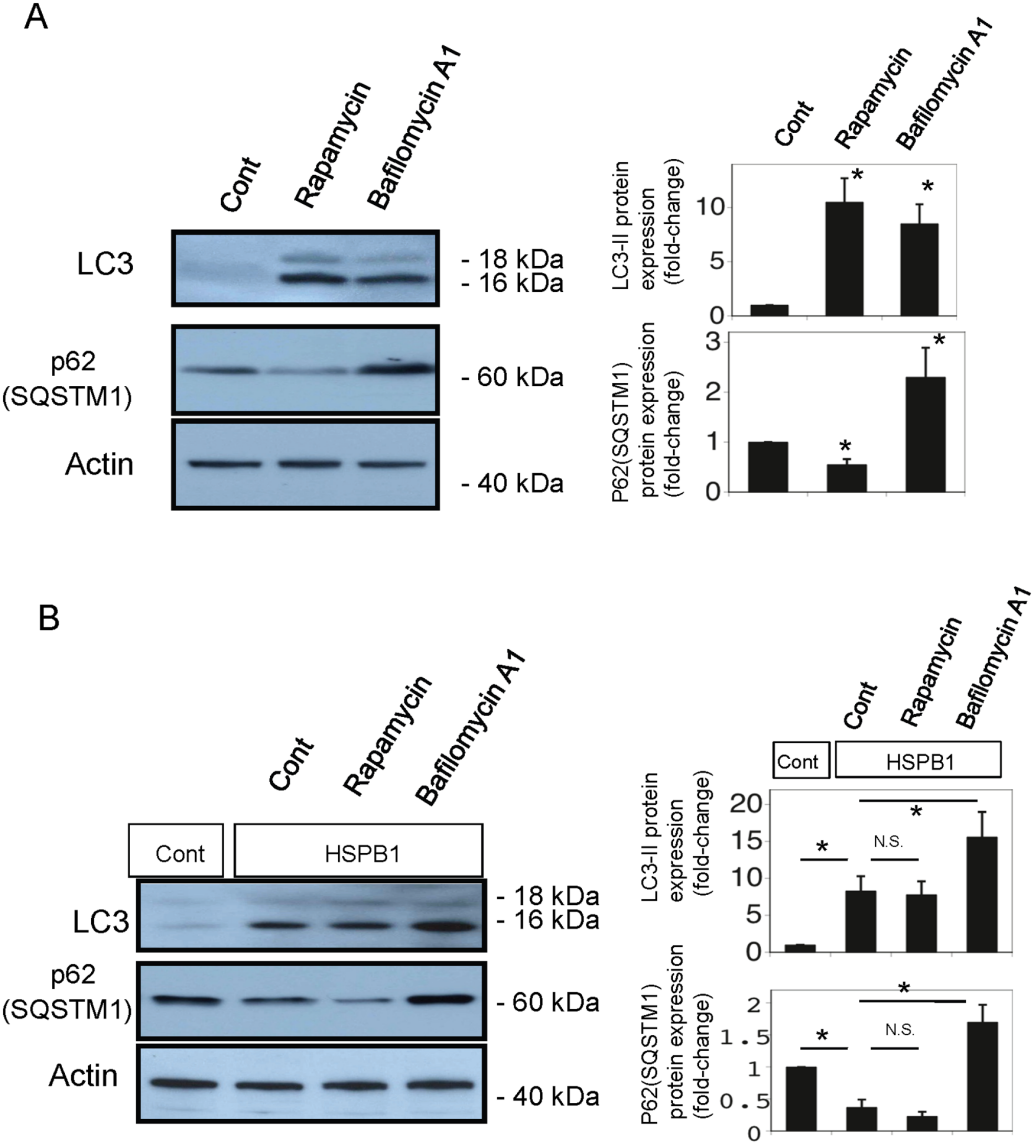
Autophagic flux is induced by overexpression of HSPB1 in NRK-52E cells. NRK-52E cells were exposed to rapamycin or bafilomycin A1. (A) Aliquots of 50 μg protein from NRK-52E cell extracts were separated with SDS-PAGE and transferred to membranes. LC3 and p62(SQSTM1) were detected with western blot analysis. Actin served as a loading control. Densitometric analysis of LC3-II protein and p62(SQSTM1) protein expression after exposure to rapamycin or bafilomycin A1. Data are presented as means ± SEM, n = 5; *P < 0.05 vs. control. NRK-52E cells transfected with HSPB1 expression vector were exposed to rapamycin or bafilomycin A1. (B) Aliquots of 50 μg protein from HSPB1-overexpressing NRK-52E cell extracts were separated with SDS-PAGE and transferred to membranes. LC3 and p62(SQSTM1) were detected with western blot analysis. Actin served as a loading control. Densitometric analysis of LC3-II protein and p62(SQSTM1) protein expression after exposure to rapamycin or bafilomycin A1. Data are presented as means ± SEM, n = 5; *P < 0.05 vs. control.

We performed experiments to differentiate between increased autophagic flux and blockade of the autophagic pathway. To monitor autophagic flux in renal tubular cells, we examined the protein expression of LC3-II and p62(SQSTM1) accumulation in cells incubated with bafilomycin A1 (200 nM, 8h) and rapamycin (100 nM, 24h). We used LC3-II and p62(SQSTM1) as markers of autophagy induction and autophagic flux, respectively. The level of LC3-II protein was increased by rapamycin and bafilomycin A1 (Fig 7A), whereas the level of p62(SQSTM1) protein was decreased by rapamycin and increased by bafilomycin A1 (Fig 7A). As shown in Fig 7B, western blot analysis showed that LC3-II protein levels were increased by HSPB1 overexpression and further elevated by bafilomycin A1. p62(SQSTM1) protein levels were decreased by the transfection of HSPB1 and increased by bafilomycin A1. These experiments suggest that overexpression of HSPB1 increased autophagic flux rather than blocking the autophagic pathway.

### Modulation of H_2_O_2_-induced apoptosis by HSPB1 overexpression in NRK-52E cells

**Fig 8:**
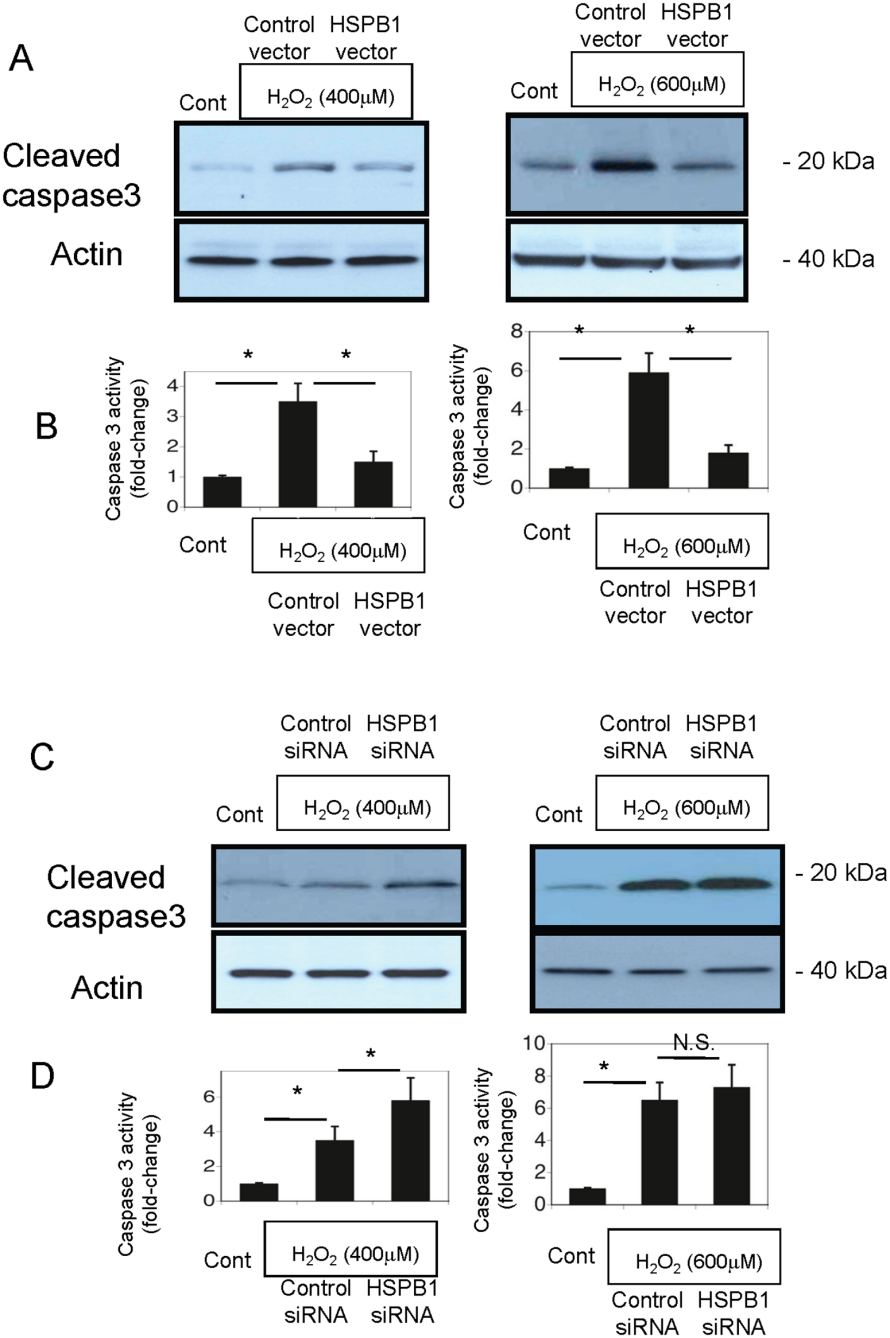
Apoptosis is reduced by HSPB1 overexpression and increased by siRNA for HSPB1 in NRK-52E cells. (A, B) Western blot analysis of cleaved caspase 3 (CASP3) expression and caspase 3 activity in control or HSPB1-overexpressing NRK-52E cells after incubation with 400 and 600 μM H_2_O_2_. (C, D) Western blot analysis of cleaved CASP3 expression and caspase 3 activity in NRK-52E cells transfected with siRNA for HSPB1 or control scrambled siRNA after incubation with 400 and 600 μM H_2_O_2_. Data are presented as means ± SEM, n = 6; *P < 0.05.

**Fig 9:**
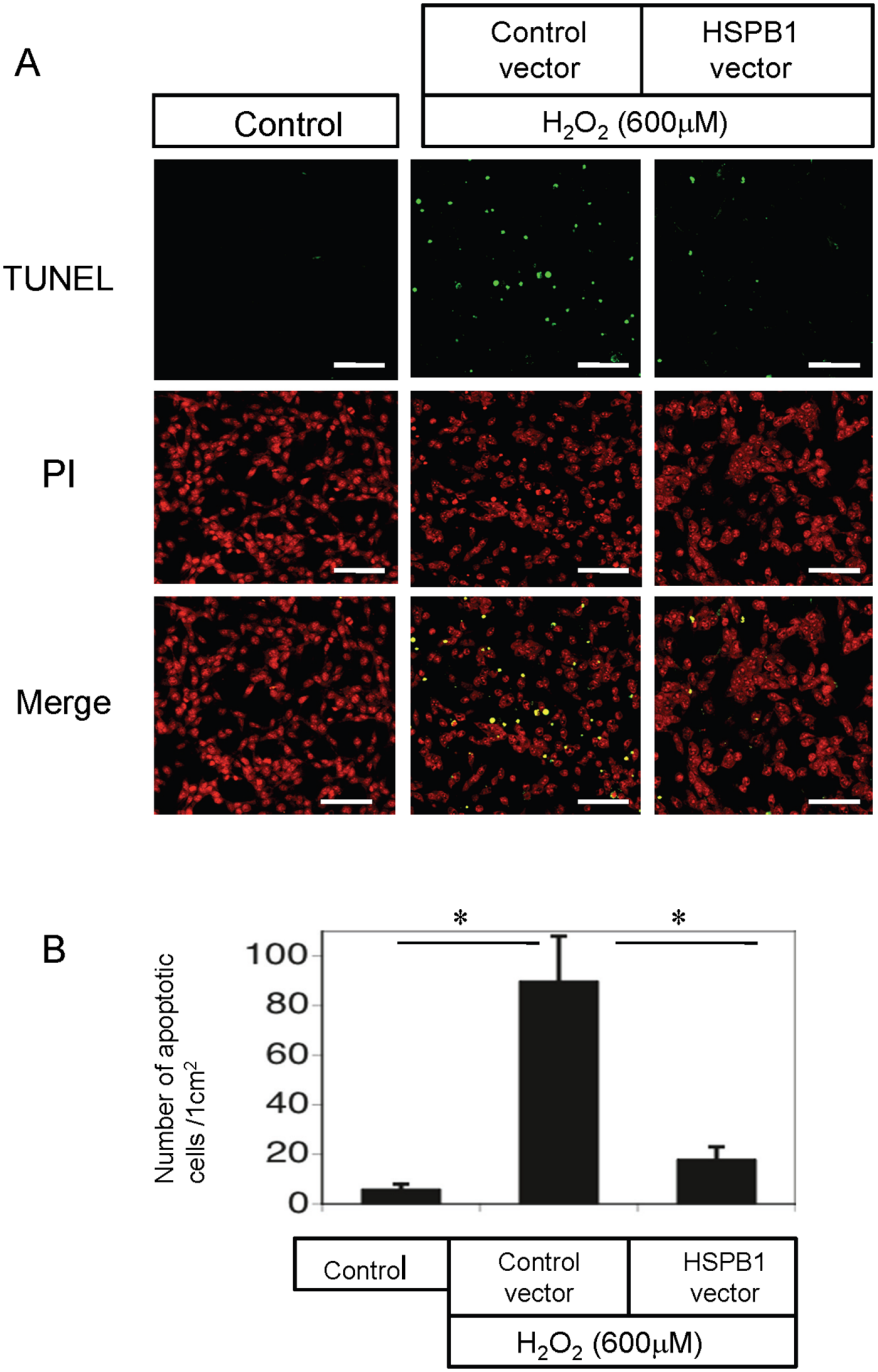
Terminal deoxynucleotidyl transferase deoxyuridine triphosphate nick end labeling (TUNEL)-positive cells are increased by oxidative stress in NRK-52E cells. (A) TUNEL assay to evaluate apoptosis in NRK-52E cells exposed to 600 μM H_2_O_2_. Nuclei were stained with propidium iodide (red). The number of apoptotic cells (green) was reduced by transfection with the HSPB1 expression vector. All scale bars represent 100 μm. (B) Quantitative analysis demonstrated that under oxidative stress induced by 600 μM H_2_O_2_, the number of apoptotic cells per square centimeter was significantly reduced by transfection with the HSPB1 expression vector. Data are presented as means ± SEM, n = 6; *P < 0.05 vs. pcDNA-transfected cells or control.

We exposed HSPB1-transfected NRK-52E cells to oxidative stress to examine the relationship between HSPB1 and apoptosis. Subsequently, we performed western blot analysis to examine the expression of cleaved caspase 3 (CASP3), a marker of apoptosis. Compared to those in the controls, the levels of cleaved CASP3 and caspase 3 activity were higher in NRK-52E cells exposed to 400 and 600 μM H_2_O_2_ for 4 h and lower in cells that overexpressed HSPB1 (Fig 8A and 8B). The levels of cleaved CASP3 and caspase 3 activity in NRK-52E cells exposed to 400 and 600 μM H_2_O_2_ for 4 h were higher in cells that overexpressed siRNA for HSPB1 (Fig 8C and 8D). The results of TUNEL staining, which detected apoptosis in NRK-52E cells under oxidative stress on exposure to 600 μM H_2_O_2_ for 4 h, showed that apoptosis was significantly reduced by transfection with the HSPB1 overexpression vector (Fig 9A and 9B). These data are consistent with the results of western blot analysis of cleaved CASP3.

### HSPB1 reduces active BAX in H_2_O_2_-treated NRK-52E cells

**Fig 10:**
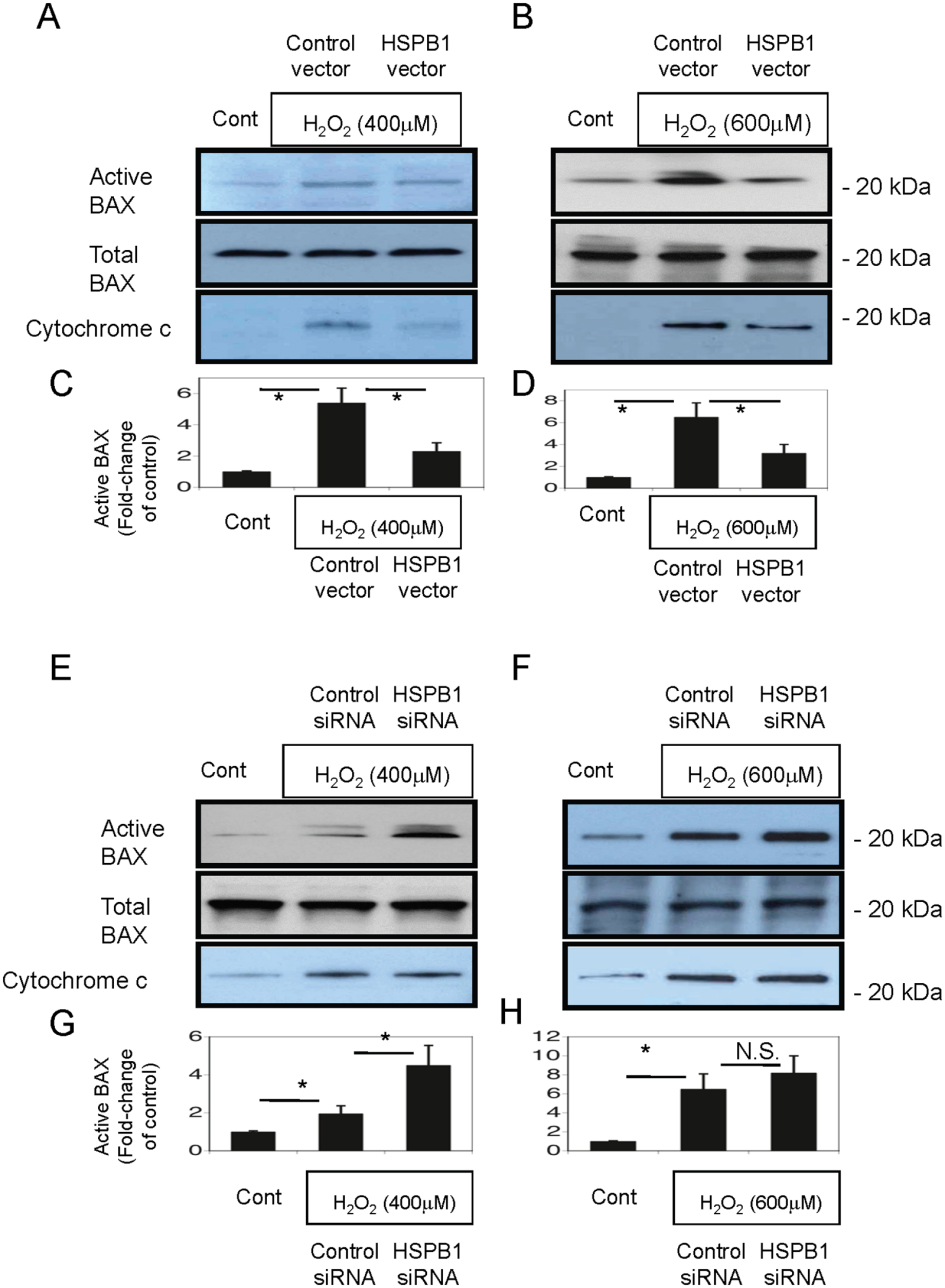
BAX activation and cytochrome c release in NRK-52E cells are reduced by overexpression of HSPB1 after H_2_O_2_ treatment. (A, B) Determination of active BAX content assessed with a conformation-specific antibody directed against the 6A7 epitope, total BAX content, and cytochrome c of cytosol fractions in control NRK-52E cells and NRK-52E cells transfected with pcDNA or the HSPB1 overexpression vector and treated with 400 and 600 μM H_2_O_2_ for 4 h. (C, D) Quantitative analysis with a densitometer showed a marked reduction in BAX activation when cells were transfected with the HSPB1 expression vector. Data are presented as means ± SEM, n = 6; *P < 0.05. (E, F) NRK-52E cells were transfected with control scrambled siRNA or HSPB1 siRNA, and the active BAX, total BAX, and cytochrome c of cytosol fractions were examined in control cells and cells exposed to 400 and 600 μM H_2_O_2_ for 4 h. (G, H) Quantitative analysis with a densitometer demonstrated that HSPB1 regulated BAX activation in NRK-52E cells. Data are presented as means ± SEM, n = 6; *P < 0.05 vs. control.

We examined the effect of HSPB1 on Bax activation because BAX is a primary cause of stress-induced mitochondrial membrane injury after oxidative stress [22, 23, 24]. Compared with baseline levels of expression, Bax expression in NRK-52E cell lysates treated with H_2_O_2_ (400 and 600 μM) for 4 h was markedly higher without changing the total BAX content in an assessment with a 6A7 epitope-specific antibody (Fig 10A, upper panel). HSPB1 overexpression markedly reduced BAX activation during oxidative stress after exposure to H_2_O_2_ for 4 h (600 μM; Fig 10A & 10B). siRNA-mediated HSPB1 knockdown markedly increased activated BAX after oxidative stress (400 μM H_2_O_2_ for 4 h; Fig 10E, upper panel) without affecting the total BAX content. Quantitative analysis with a densitometer demonstrated that HSPB1 regulated BAX activation in NRK-52E cells (Fig 10C, 10D, 10G, and 10H). We also investigated cytoplasmic cytochrome c levels as a marker of the initial step of apoptosis triggered by the mitochondria. H_2_O_2_ treatment for 4 h markedly increased cytochrome c levels in NRK-52E cell cytoplasmic fractions. Overexpression of HSPB1 reduced oxidative stress-induced cytoplasmic cytochrome c levels, as shown in Fig 10A and 10B. siRNA-mediated HSPB1 knockdown induced oxidative stress-induced cytoplasmic cytochrome c levels, as shown in Fig 10E and 10F.

### HSPB1 reduces endoplasmic reticulum stress in H_2_O_2_-treated NRK-52E cells

**Fig 11:**
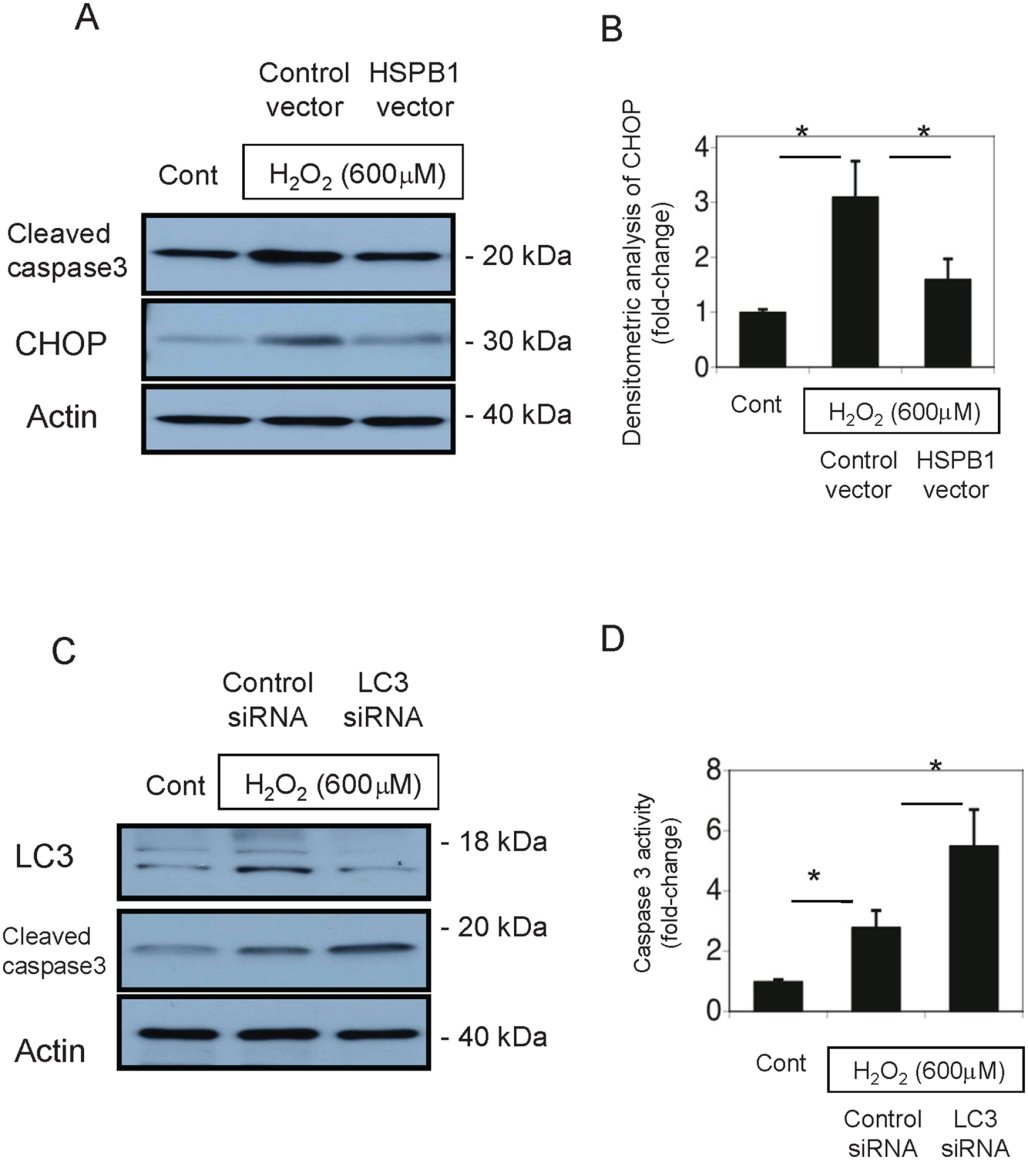
HSPB1 reduced an endoplasmic reticulum (ER) stress marker in H_2_O_2_-treated NRK-52E cells, and inhibition of LC3 increased cleaved CASP3. (A) NRK-52E cells were transfected with a control vector or HSPB1 expression vector, and the expressions of cleaved CASP3 and CHOP were examined after exposure to 600 μM H_2_O_2_ for 4 h. (B) Quantitative analysis with a densitometer demonstrated that HSPB1 reduced CHOP expression in NRK-52E cells. (C) NRK-52E cells were transfected with control siRNA or siRNA for LC3. The expressions of LC3 and cleaved CASP3 were examined in cells exposed to 600 μM H_2_O_2_ for 4 h. (D) Caspase 3 activity in NRK-52E cells transfected with siRNA for LC3 or control scrambled siRNA after incubation with 600 μM H_2_O_2_ for 4 h. Data are presented as means ± SEM, n = 6; *P < 0.05.

We examined the effects of HSPB1 on oxidative stress-induced endoplasmic reticulum (ER) stress in renal tubular cells. H_2_O_2_ (600 μM) treatment for 4 h markedly increased the expression of the ER stress marker CHOP in NRK-52E cell lysates. Overexpression of HSPB1 reduced oxidative stress-induced CHOP expression, as shown in Fig 11A and 11B.

### Inhibition of autophagy induces caspase 3 activation in H_2_O_2_-treated NRK-52E cells

We performed experiments using siRNA for LC3 to determine the effects of autophagy on apoptosis. The transfection of siRNA for LC3 markedly reduced LC3 expression in NRK-52E cells. This inhibition of LC3 by siRNA increased cleaved CASP3 after H_2_O_2_ (600 μM for 4 h) treatment (Fig 11C and 11D).

## Discussion

Two novel findings arose from this study. First, we showed that HSPB1 expression increases in renal proximal tubules during AKI *in vivo* and that oxidative stress induces promoter activity as well as mRNA and protein expression in NRK-52E cells. Second, we demonstrated that HSPB1 expression causes autophagy and inhibits apoptosis in renal tubular cells. Hence, our findings indicate that HSPB1 may protect renal tubules in AKI. To the best of our knowledge, this study is the first to demonstrate the induction of autophagy by HSPB1 and its inhibition by HSPB1 siRNA in renal tubular cells. Relatively few studies have described the role of HSPB1 in the prevention of apoptosis and cell death via autophagy. A recent study of a hepatocellular carcinoma cell line showed that HSPB1 protects cells from cisplatin-induced death through an autophagy mechanism [25], and suggested a connection between autophagy-related gene 7 and HSPB1. Recent publications have revealed the role of the high-mobility group box 1 (HMGB1)-HSPB1 pathway in the regulation of autophagy/mitophagy [26, 27], demonstrating that HMGB1 regulates HSPB1 expression and is essential in the regulation of autophagy and mitochondrial quality control in mouse embryonic fibroblasts. To the best of our knowledge, no studies have elucidated the relationship between HSPB1 and autophagy in renal tubular cells. We have clearly demonstrated that HSPB1 overexpression induces autophagy and that HSPB1 siRNA inhibits H_2_O_2_-induced autophagy in NRK-52E cells. HMGB1 is reportedly upregulated in a septic AKI model [28]. Further investigation of the relationship between HMGB1 and HSPB1 in autophagy in an I/R AKI model is warranted. Park et al. [29] and Chen et al. [30] reported that although the induction of HSPB1 in renal tubular cells protects against necrosis *in vitro*, its systemic increase counteracts this protection by exacerbating renal and systemic inflammation *in vivo*. The functional roles of HSPB1 in AKI warrant further investigation.

We previously used GFP-LC3 transgenic mice to investigate autophagy in kidney tissues during cisplatin-induced nephrotoxicity and demonstrated that autophagy occurs mainly in the proximal tubules [4]. In addition, we recently reported that at least two pathways, p53-sestrin2 and HIF-1-BNIP3, are involved in the induction of autophagy in the renal tubules during AKI [21]. The results of the current study are consistent with the findings of these previous studies and revealed that HSPB1 is another mediator of autophagy in AKI. We performed experiments to differentiate between increased autophagic flux and blockade of the autophagy pathway using LC3-II and p62(SQSTM1) as markers of autophagy induction and autophagic flux, respectively. As shown in Fig 7, western blot analysis showed that LC3-II protein levels were increased by HSPB1 overexpression and further elevated by rapamycin. p62(SQSTM1) protein levels were decreased by the transfection of HSPB1 and increased by bafilomycin A1. Therefore, the results of these experiments suggest that overexpression of HSPB1 increased autophagic flux rather than blocking autophagy.

The role of autophagy in the pathogenesis of AKI is still under debate, although most pharmacological and genetic studies support a renoprotective role for autophagy in renal tubular cells. Several studies have indicated that autophagy is induced as part of an adaptive response that reduces apoptosis and prolongs the survival of renal tubular cells [31, 32]. This conclusion is consistent with the finding that renal I/R injury is exacerbated when autophagy is chemically or genetically inhibited. Other studies have concluded that autophagy is a protective mechanism for cell survival [6, 7].

Many cytoprotective roles have been reported for the HSP family [12, 13]. However, relatively little information is available regarding the sHSP subfamily, which includes HSPB1. Fujigaki et al. [33] demonstrated that HSPB1 accumulates in proximal tubules subjected to acute tubular injury, and described the possibility that it contributes to the survival and regeneration of proximal tubular cells. In the present study, we observed slight upregulation of phosphorylated HSPB1 in sham-operated rats, in which changes in blood pressure or oxygen saturation may occur under anesthesia. The induction of HSPB1 phosphorylation is much more dramatic in I/R-injured rats. We demonstrated that HSPB1 regulates both autophagy and apoptosis in renal tubular cells. The role of HSPB1 was first described by Landry et al. [34], who demonstrated that HSPB1 protects cells from thermal stress.

Later, the cytoprotective effect of HSPB1 against various apoptotic effectors was demonstrated. Using renal cells, Sanchez-Nino et al. [35] demonstrated that HSPB1 downregulation by siRNA increases the podocyte apoptosis rate *in vitro*. Previous reports have shown that HSPB1 also has a neuroprotective effect [36, 37] and that HSPB1 overexpression reduces apoptosis induced by ischemia in the liver [38]. Furthermore, HSPB1 regulates apoptosis in polymorphonuclear leukocytes [39]. In myeloma cell lines, HSPB1 activation reportedly protects cells from apoptosis by blocking the mitochondrial release of the second mitochondria-derived activator of caspase [20, 40].

The signal transduction pathway between HSPB1 and apoptosis is controversial. In renal epithelial cells under stress conditions, HSPB1 indirectly inactivates Bax and its translocation to the mitochondria [22]. This inactivation occurs because an increase in PI3-kinase activity activates Akt, a pro-survival kinase, and promotes interactions between Akt and Bax. In this study, we clearly showed that overexpression of HSPB1 reduced the activation of BAX in NRK-52E cells. These data are consistent with those of previous reports [22]. HSPB1 could indirectly regulate Bax by altering the activity of kinases, including Akt and GSK3β, by which Bax is phosphorylated [22, 41, 42]. Furthermore, we showed that overexpression of HSPB1 reduced ER stress markers. These data indicate that the anti-apoptotic pathway of HSPB1 is mediated, at least in part, by Bax and the regulation of ER stress. The results of the present study and others, therefore, show that HSPB1 inhibits apoptotic cell death [22, 35]. We found that oxidative stress-induced apoptosis is ameliorated by HSPB1 in renal tubular cells, and this finding may be indicative of a new therapeutic role for HSPB1 in AKI.

One limitation of this study is that our *in vivo* experiments were undertaken to investigate the role of HSPB1 in autophagy and apoptosis only during oxidative stress. Treatment with H_2_O_2_ is one of the many *in vivo* models of AKI. Glucose withdrawal and transient hypoxia are other available models. In future studies, we will use these models to further investigate the role of HSPB1 in AKI.

In summary, we demonstrated that HSPB1 expression is increased in the renal proximal tubules in a rat model of AKI, and that incrementally increasing HSPB1 expression causes autophagy and inhibits apoptosis in renal tubular cells. These results indicate that upregulation of HSPB1 plays a role in the pathophysiology of AKI.
